# Metachronous Human Papillomavirus (HPV)-Related Eye Carcinoma in Previously Metastatic Breast Cancer: A Case Report

**DOI:** 10.7759/cureus.64198

**Published:** 2024-07-09

**Authors:** Jorge Sanchez, Nehemias Guevara, Yemesrach Mekonen, Azana Newman, Volha Chapiolkina, Esmirna M Perez Rosario, Somayeh Fahim, Ilmana Fulger

**Affiliations:** 1 Internal Medicine, St. Barnabas Hospital Health System, New York, USA; 2 Hematology-Oncology, St. Barnabas Hospital Health System, New York, USA

**Keywords:** breast cancer, hpv carcinoma, metastatic breast cancer, eye carcinoma, metachronous cancers

## Abstract

Multiple primary malignancies (MPMs) occur when an individual develops two or more distinct primary cancers. These are categorized as synchronous or metachronous based on the timing of their diagnosis. Patients previously diagnosed with cancer face increased risks due to exposure to carcinogenic factors and treatments such as chemotherapy and radiotherapy.

Individuals with a history of breast cancer are known to have elevated risks for secondary malignancies compared to the general population. However, cases of squamous cell carcinoma (SCC) of the eyelid in this group are exceedingly rare. Here, we present a case report describing a young female patient who sequentially developed metachronous breast cancer, and a human papillomavirus (HPV)-associated SCC of the eyelid.

To the best of our knowledge, this case report represents the first documented instance of this specific combination of primary neoplasms in medical literature.

## Introduction

Multiple primary malignancies (MPMs) refer to the occurrence of two or more separate primary cancers in an individual. Based on the timing of tumor diagnoses, MPM can be categorized into synchronous and metachronous types. Synchronous MPM refers to cases where a second tumor is diagnosed simultaneously or within two months of the first tumor, whereas metachronous MPM involves the identification of a second tumor more than six months after the first tumor [1].

MPM is often encountered in linked organs, such as the breast, ovaries, and the colon, commonly associated with MPM [2]. Breast cancer is the most common type of cancer among women and ranks among the leading causes of cancer-related deaths [3]. Patients with a history of breast cancer are at a higher risk of developing secondary malignancy compared to the general population [4]. Secondary malignancies can develop as a result of either past medical treatments (like radiation or chemotherapy) or certain habits (such as smoking and drinking alcohol). It is essential to differentiate MPM from metastases and secondary cancers. In this report, we present an unusual case of metachronous breast cancer and squamous cell carcinoma (SCC) of the eyelid in the same patient.

## Case presentation

A 34-year-old female who initially presented to the doctor after feeling a right breast mass had imaging at the time (breast ultrasound and mammography), which showed a right breast mass measuring 2.4 cm with a breast imaging-reporting and data system (BI-RADS) of 5, representing a high suggestion of malignancy. Ultrasound-guided core biopsy showed moderately differentiated invasive ductal carcinoma (IDC), estrogen receptor (ER) positive in >90%, progestin receptor (PR) positive in >90%, and human epidermal growth factor receptor 2 (HER2) positive by immunohistochemistry (IHC), but fluorescence in situ hybridization (FISH) was negative.

The patient elected for a bilateral mastectomy with immediate reconstruction; pathology showed right breast IDC measuring 3.8 cm, moderately differentiated, with associated ductal carcinoma in situ (DCIS) solid and cribriform type with necrosis, intermediate nuclear grade, all margins negative. The breast cancer gene test (BRCA) was negative. No axillary nodes were found. 

A couple of months later, the patient had a sentinel lymph node biopsy (SLNB) negative for carcinoma. She was started on adjuvant ovarian suppression with monthly leuprolide and tamoxifen. She experienced several side effects, like sleepiness, fatigue, and mood swings, in the first three weeks of treatment with tamoxifen, making it intolerable for her, and was subsequently changed to letrozole.

**Figure 1:**
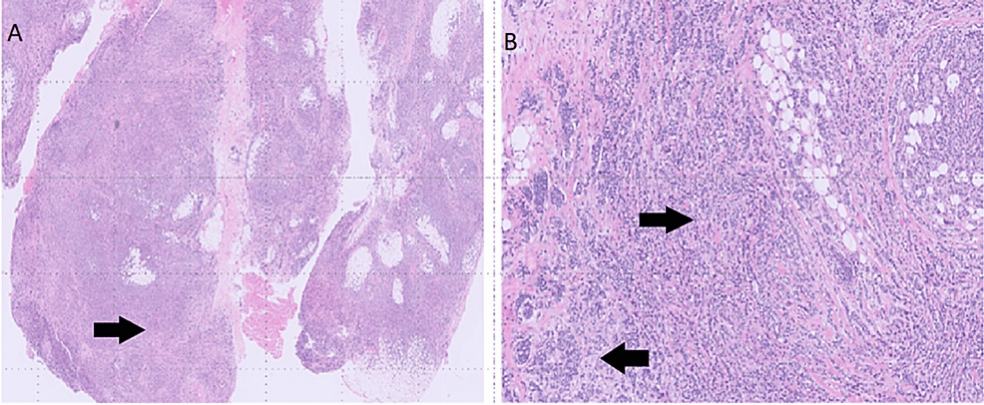
Histological examination reveals A) fibro-adipose tissue infiltrated by a poorly differentiated carcinoma (indicated by the arrow) on the left slide. B) Under low magnification, nests and cords of cells (indicated by the arrows) are consistent with metastatic carcinoma compatible with primary breast cancer. Both slides were stained with hematoxylin and eosin (H&E).

Two years later, she developed progressive left exophthalmos and was found to have a retrobulbar mass on imaging. She had a biopsy of the mass that confirmed recurrent metastatic carcinoma compatible with primary breast cancer (Figure 1).

Histology showed fibro-adipose tissue involved by a poorly differentiated carcinoma infiltrating in a single file pattern and small nests. Tumor cells were positive for cytokeratin 7 (CK7) and GATA3 protein; there was negative staining for cytokeratin 20 (CK20) and mammaglobin. Also, the tumor was >95% positive for ER and less than 1% positive for PR; HER2 was negative.

Positron emission tomography (PET/CT) showed a hypermetabolic left retro-orbital mass, hypermetabolic retropharyngeal lymphadenopathy, focal hypermetabolic uptake of the right breast centrally concerning for recurrence, and multiple areas of focal bony uptake in the right posterior acetabulum, left iliac wing, and left sacrum. The patient received radiotherapy to the left retrobulbar mass and re-started leuprorelin monthly for ovarian suppression with letrozole/palbociclib. She underwent elective bilateral oophorectomy one year later with benign findings, and for two years, she was clinically and radiologically with an excellent response. 

Four years after the initial breast cancer diagnosis, she started developing a mass in the right eyelid with persistent tearing and discomfort. Concern for another site of metastasis was raised, especially because she already had metastatic disease in her left eye. Therefore, a biopsy was done; however, surprisingly, the biopsy showed a different type of cancer.

The biopsy revealed a carcinoma with squamous differentiation, featuring focal areas of sebaceous elements that were highlighted using an adipophilin immunostain. The cells displayed pleomorphism and marked atypia. Low-risk human papillomavirus (HPV) types (6 and 11) tested negative, while high-risk HPV types (16 and 18) showed strong positivity through in situ hybridization.

**Figure 2:**
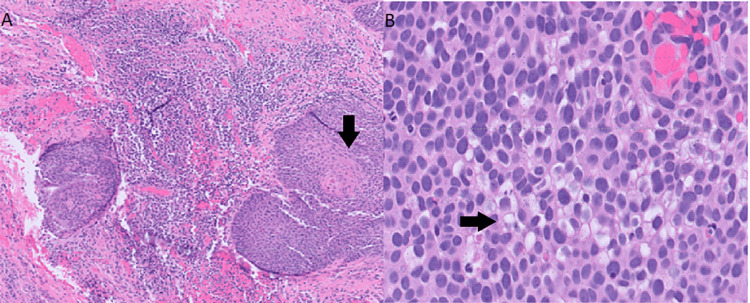
A) Invasive squamous cell carcinoma exhibits areas of keratinization (indicated by the arrow); B) focal areas of sebaceous differentiation (also indicated by the arrow) and no gland formation, which is characteristic of HPV-related SCC. Both slides were stained with hematoxylin and eosin (H&E). HPV: Human papillomavirus; SCC: Squamous cell carcinoma

These findings, consistent with HPV-related invasive SCC (Figure 2), confirmed the diagnosis.

PET/CT was done and did not reveal any new site of possible metastasis or nodal involvement.

Based on the diagnosis and stage (stage I), she was scheduled to be treated with surgical resection followed by topical 5-fluorouracil and radiotherapy. Currently, the patient is under chemotherapy treatment with a good response.

## Discussion

The case described here meets the criteria for metachronous cancer. Specifically, the patient presented with two primary and histologically distinct cancers in different organs: HPV-positive SCC of the skin on the eyelid and infiltrating ductal carcinoma of the breast. Since each of these cancers is considered a primary neoplasm, a diagnosis of metachronous primary cancer was determined. Metachronous neoplasms are defined as two or more primary neoplasms diagnosed after an interval of more than six months, with each neoplasm being a distinct malignancy rather than a metastasis of the other [1]. 

HPVs are small double-stranded oncogenic viruses [5], and it is the most common sexually transmitted disease, with a worldwide prevalence of 32% among women [6]. Infection with anogenital HPV typically does not cause symptoms and often resolves on its own without consequences in individuals with a healthy immune system. However, when symptoms do appear, the most frequent manifestation is genital warts, which can vary in appearance from small papules to flat, smooth, or pedunculated lesions [7,8].

The development of malignancies in the anogenital region is most commonly associated with prior infection by high-risk, oncogenic strains of HPV. In immunocompetent individuals, more than 99% of cervical cancers, 85% of anal cancers, and approximately 50% of cancers affecting the vulva, vagina, and penis are attributed to oncogenic HPV strains [7,8].

Scientific studies have discussed the role of HPV in squamous cell neoplasia of the ocular adnexa, but findings have been inconclusive due to the rarity of these cancers and limited research. Recent studies indicate that certain types of conjunctival papillomas, intraepithelial neoplasia, and carcinomas may develop through an HPV-dependent pathway. However, the precise involvement of HPV in squamous cell tumors occurring in the lacrimal drainage system and eyelid remains unclear [9].

Patients with a history of cancer are at higher risk of developing additional neoplasms due to exposure to common carcinogens like tobacco and alcohol, genetic predisposition, or as a consequence of previous chemotherapy or radiotherapy. In the present case, the likely contributing factor was HPV infection, as the patient had previously tested positive for HPV during Pap smear co-testing. According to Gallagher et al., exposure to insecticides, herbicides, fungicides, or seed treatments has also been linked to an increased incidence of skin SCC [10]. Furthermore, Pastore et al. documented a case where an elderly woman had synchronous ureteral and bladder metastases originating from infiltrating ductal breast carcinoma [11]. It is thought that the emergence of multiple primary neoplasms arises from several factors, such as enhanced cancer survival rates and extended lifespans, which provide more time for the development of additional cancers [5].

SCC of the eyelid is relatively uncommon in the literature [12], accounting for only approximately 5-10% of all eyelid carcinomas [13]. Eyelid SCC may initially present as a non-healing mass with distinct characteristics, including small red plaques and papillomatous, nodular, or ulcerative lesions. Growths are typically painless and progress gradually. Growth of tumors may lead to obstruction of vision, diplopia, eye pressure, and even increased intraocular pressure. Early-stage eyelid SCC may sometimes mimic chronic anterior blepharitis, leading to potential misdiagnosis. Standard treatment usually consists of surgical resection. Other treatments include radiotherapy and chemotherapy, including the ophthalmic solutions of imiquimod, 5-fluorouracil, and interferon alpha-2b [14]. In this patient's case, the plan was made for surgical excision and treatment with 5-fluorouracil and possible radiotherapy.

## Conclusions

Our case highlights the importance of vigilant monitoring for secondary malignancies in cancer survivors. The discovery of metachronous HPV-related SCC of the eyelid in a patient previously diagnosed with metastatic breast cancer emphasizes the need for comprehensive surveillance and tailored treatment strategies. Healthcare providers must prioritize follow-up plans and lifestyle modifications to mitigate risks and enhance the overall well-being of survivors.
